# The Early Implementation of a Hub-and-Spoke Survivorship Pathway for Out-of-Hospital Cardiac Arrest Survivors: A 12-Month Formative Evaluation of the REVIVE Project

**DOI:** 10.3390/jcm15124722

**Published:** 2026-06-17

**Authors:** Laura Calabrese, Marco Mion, Alice Mandrini, Roberto Primi, Sara Bendotti, Leila Ulmanova, Alessia Currao, Arianna Morena, Filippo Dossi, Leonardo Fogagnolo, Federica Pizzi, Cristian Fava, Daniele Ghiraldin, Alessio Battioni, Paola Genoni, Elena Maria Paola Madonini, Diego Maffeo, Cinzia Dossena, Silvia Affinito, Giovanni Bertazzoli, Marta Pellegrino, Gioele Papi, Silvia Frattini, Matteo Della Torre, Cecilia Fantoni, Angelica Praderio, Luca Tarantino, Salvatore Mongiovì, Pierluigi Politi, Simone Savastano, Enrico Baldi, All the LombardiaCARe Researchers

**Affiliations:** 1 Division of Cardiology, Fondazione IRCCS Policlinico San Matteo, 27100 Pavia, Italy; l.calabrese@smatteo.pv.it (L.C.); m.mion@nhs.net (M.M.); alice.mandrini@gmail.com (A.M.); r.primi@smatteo.pv.it (R.P.); s.bendotti@smatteo.pv.it (S.B.); l.ulmanova@smatteo.pv.it (L.U.); a.currao@smatteo.pv.it (A.C.); ariannamorena0@gmail.com (A.M.); s.savastano@smatteo.pv.it (S.S.); 2 Cardiac Arrest and Resuscitation Research Team (RESTART), Fondazione IRCCS Policlinico San Matteo, 27100 Pavia, Italy; 3 Anglia Ruskin School of Medicine & MTRC, Anglia Ruskin University, Chelmsford CM1 1SQ, UK; 4 Department of Mental Health and Addiction Services, ASST Pavia, 27100 Pavia, Italy; 5 Department of Public Health, Experimental and Forensic Medicine, University of Pavia, 27100 Pavia, Italy; 6 Department of Internal Medicine and Medical Therapy, University of Pavia, 27100 Pavia, Italy; 7 Department of Molecular Medicine, University of Pavia, 27100 Pavia, Italy; 8 Ospedale di Circolo e Fondazione Macchi di Varese, ASST Sette Laghi, 21100 Varese, Italy; filippo.dossi@asst-settelaghi.it (F.D.); leofoga99@gmail.com (L.F.); pizzi.federica@hotmail.it (F.P.); 9 Ospedale Carlo Poma, ASST di Mantova, 46100 Mantova, Italy; cristian.fava@asst-mantova.it; 10 Ospedale Spedali Civili, ASST Brescia, 25123 Brescia, Italybattionia96@gmail.com (A.B.); 11 Ospedali Sant’Anna di Como e Sant’Antonio Abate di Cantù, ASST Lariana, 22100 Como, Italy; paola.genoni91@gmail.com (P.G.); elena.madonini@asst-lariana.it (E.M.P.M.); 12 Ospedale Poliambulanza, Fondazione Poliambulanza, 25124 Brescia, Italy; diego.maffeo@poliambulanza.it; 13 Ospedale Maggior di Crema, ASST di Crema, 26013 Crema, Italy; cinzia.dossena@asst-crema.it; 14 Ospedale di Legnano, ASST Ovest Milanese, 20025 Legnano, Italy; silviaaffinito@gmail.com; 15 Ospedale Maggiore di Lodi, ASST Lodi, 26900 Lodi, Italy; giovanni.bertazzoli@asst-lodi.it (G.B.); marta.pellegrino@asst-lodi.it (M.P.); 16 Ospedale di Cremona, ASST Cremona, 26100 Cremona, Italy; gioele.papi@asst-cremona.it (G.P.); silvia.frattini@asst-cremona.it (S.F.); matteo.dellatorre@asst-cremona.it (M.D.T.); 17 Ospedale Humanitas Mater Domini, 21053 Castellanza, Italy; cecilia.fantoni@mc.humanitas.it; 18 Ospedale di Manerbio, ASST Garda, 25025 Manerbio, Italy; angelica.praderio@asst-garda.it; 19 Ospedale di Chiari, ASST Franciacorta, 25032 Chiari, Italy; luca.tarantino@asst-franciacorta.it; 20 Ospedali di Busto Arsizio e Gallarate, ASST Valle Olona, 21052 Busto Arsizio, Italy; salvo.mongiovi@gmail.com; 21 Department of Brain and Behavioural Sciences, University of Pavia, 27100 Pavia, Italy; pierluigi.politi@unipv.it

**Keywords:** health status assessment, out-of-hospital cardiac arrest, patient outcome assessment, quality of life, survivorship, barriers, facilitators

## Abstract

**Background/Objectives:** A structured follow-up after out-of-hospital cardiac arrest (OHCA) is recommended, but implementation across regional networks remains challenging. REVIVE introduced a hub-and-spoke survivorship pathway in Lombardy. This 12-month formative implementation evaluation aimed to describe staged pathway progression, operational reach, attrition points, centre-level variation, and documented barriers to assessment completion. **Methods:** Adult OHCA survivors with Cerebral Performance Category (CPC) 1–2 or Modified Rankin Scale (mRS) ≤ 3 were considered eligible. The evaluation was structured using Proctor et al.’s implementation outcomes framework. Implementation outcomes were operationalised using prospectively collected pathway indicators: eligibility ascertainment, successful contact, T0 assessment completion, completion of planned assessment components, timeliness where available, and documented reasons for non-progression. Analyses were descriptive and used chi-square or Fisher’s exact tests for unadjusted centre-level comparisons. **Results:** Of the 1663 patients hospitalised, 1458 (87.7%) were recorded as deceased or having an unfavourable neurological outcome and were therefore outside the intended REVIVE target population. Among the remaining 205 patients, eligibility could not be determined for 78 (4.7% of the total cohort), and 127 (7.6%) met eligibility criteria. Of eligible survivors, 96 (75.6%) were contacted and 64 completed the T0 assessment (66.7% of contacted; 50.4% of eligible). Pavia showed higher observed rates of eligibility ascertainment, contact, and assessment completion than spoke centres, but these differences were unadjusted and should be interpreted as centre-level implementation variation rather than evidence of causal superiority. **Conclusions:** REVIVE initiated a structured regional pathway for post-OHCA follow-up, but first-year implementation was partial rather than definitive. The 50.4% T0 completion rate among eligible survivors should be interpreted as an initial internal implementation indicator, not as evidence of established feasibility, effectiveness, or regional benchmarking. Priorities for further optimisation include eligibility ascertainment, transfer of contact information, patient engagement, and spoke-site support for assessment delivery.

## 1. Introduction

Survivorship after out-of-hospital cardiac arrest (OHCA) represents an increasingly important area of focus across services involved in the care of patients recovering from critical illness [1]. Advances in pre-hospital and in-hospital care have led to an increase in the number of survivors; part of the focus is therefore now shifting to long-term quality of life, as many of them experience persistent cognitive deficits, emotional distress, fatigue, functional limitations, and reduced participation in daily activities [2,3]. These multidimensional aspects are often under-recognised in routine cardiology follow-up and support for survivors and their relatives are frequently fragmented or absent [4,5].

International guidelines now recommend structured, multidisciplinary follow-up for cardiac arrest survivors and their families, with the systematic assessment of physical, cognitive, and psychological outcomes and the provision of tailored information and rehabilitation [1]. Published survivorship pathways report coverage of 30–50% eligible survivors, primarily as single-centre models [6] or regional audits without structured coordination [7]. A local example of a structured survivorship programme has been reported, describing the implementation of a multidisciplinary follow-up pathway for OHCA survivors, integrating cardiac rehabilitation, psychological support, and patient education, with early signals of feasibility and potential clinical benefits [6]. However, practical gaps in the implementation of European Resuscitation Council (ERC) guidelines are still likely to persist, even in centres offering a similar service, with only partial adoption of survivorship follow-up practices [7].

The REVIVE project was created to operationalise these recommendations through a hub-and-spoke survivorship pathway, using a specialist coordinating centre to support participating hospitals [8]. In the present paper, REVIVE is treated as a service-delivery intervention whose early objective lies in identifying how reliably eligible survivors can be identified, contacted, and assessed across a regional network, rather than in demonstrating clinical effectiveness or survivor outcomes.

To characterise implementation processes and identify optimisation opportunities, we conducted a formative implementation evaluation structured using Proctor et al.’s taxonomy of implementation outcomes [9]. This framework was selected because the available data primarily describes implementation processes, including reach, feasibility, fidelity and acceptability, rather than clinical effectiveness. The primary aims of this study were to evaluate the early implementation of the REVIVE survivorship follow-up pathway during its first 12 months of delivery across participating centres. Specifically, the study examined staged pathway progression, eligibility ascertainment, successful contact, T0 assessment completion, centre-level variation, and documented reasons for non-entry, non-completion, and drop-out. A secondary aim was to identify practical optimisation targets for future implementation cycles before evaluating longer-term feasibility, acceptability, sustainability, and survivor outcomes.

## 2. Methods

The REVIVE project operationalises a sub-regional “hub-and-spoke” model in which a specialised cardiac arrest centre (“hub”) provides coordinated post-discharge assessment and follow-up for survivors admitted to participating hospitals (“spokes”). The pathway includes the early post-discharge (T0) assessment of cognitive function, psychological symptoms, and health-related quality of life using standardised instruments (e.g., Montreal Cognitive Assessment, MoCA; Hospital Anxiety and Depression Scale, HADS; Impact of Event Scale-Revised, IES-R; EQ-5D-5L), and through a semi-structured interview. These domains are reassessed at periodic follow-ups at one month (T1), three months (T2), six months (T3), and one year (T4) after the event. Assessments are offered in multiple modalities: in person, online, and by telephone.

This study used a prospective formative implementation evaluation design to assess the early delivery of the REVIVE pathway during its first 12 months of delivery (January 2025–December 2025) across participating centres in Lombardy, Italy. Detailed methods for the broader REVIVE protocol, eligibility criteria, assessment tools, and follow-up schedule are described in the published protocol. The present analysis focuses on the implementation progression and operational performance rather than clinical effectiveness [8].

### 2.1. Implementation Evaluation Framework

The evaluation used Proctor et al.’s implementation outcomes framework to define and structure the implementation indicators. The first-year report focused on domains that could be measured from the prospective REVIVE database: reach/penetration, feasibility, fidelity, and acceptability. Adoption was described at centre level through participation in the pathway and the use of local referents. Appropriateness, implementation cost, sustainability, and clinical effectiveness were not formally evaluated in this first-year analysis.

Reach/penetration was operationalised as the proportion of patients for whom eligibility could be determined, the proportion of eligible survivors contacted, and the proportion completing T0 assessment. Feasibility was operationalised as the successful delivery of the staged pathway across participating centres. Fidelity was operationalised as the completion of planned assessment components and timeliness where available. Acceptability was operationalised pragmatically through documented refusal, non-response, lack of interest, perceived well-being, overload, and logistical barriers.

### 2.2. Study Centres and Eligibility Determination

Participating centres included one university-affiliated tertiary “hub” hospital (Policlinico San Matteo, Pavia) and 21 “spoke” hospitals (18 non-university and 3 university centres) across the Lombardy region. University hospitals were defined as centres with formal academic affiliation, teaching status, and structured undergraduate/postgraduate medical training programmes, while non-university hospitals lacked these characteristics.

All consecutive adult OHCA survivors admitted to participating hospitals (January–December 2025) were prospectively screened for eligibility by local cardiologists using the following standardised protocol criteria:

*Inclusion criteria*: Patients aged ≥ 18 years who survived an out-of-hospital cardiac arrest (OHCA) with resuscitation attempted by the Emergency Medical System, showing good neurological outcome (Cerebral Performance Category [CPC] 1–2 or Modified Rankin Scale [mRS] ≤ 3 at discharge), capable of providing written informed consent.

*Exclusion criteria*: Patients unable to speak the language sufficiently to participate in assessments, or with acute unresolved psychological disorders of moderate/severe intensity (e.g., major depression, schizophrenia).

Eligible survivors were assessed by local clinicians (hub/spoke cardiologists), informed about the REVIVE project, and provided informed consent if interested. Weekly reminders from the Pavia hub team to spoke clinicians aimed to minimise missed cases. Following consent, patient contact details were forwarded to the clinical psychologist, who scheduled the T0 assessment: in-person at Pavia hub or remotely (telephone/telemedicine) for spoke centres. A prospective cohort flow-chart tracked patient progression through pathway stages: screening, eligibility determination, consent, hub team contact, and T0 assessment completion. Each stage and the reasons for non-progression were recorded prospectively in the REVIVE database. These data provided the indicators for the Proctor implementation outcomes described above. Barriers were identified from documented attrition reasons and recurring implementation failures visible in pathway tracking rather than from formal qualitative interviews; they should therefore be interpreted as operational barriers.

### 2.3. Statistical Analysis

Categorical variables were summarised as counts and percentages and compared between centre types using chi-squared or Fisher’s exact tests (for expected cell counts < 5). Continuous variables were summarised as mean ± standard deviation (SD) or median and interquartile range (IQR), based on distribution assessed via Shapiro–Wilk normality tests. All tests were two-sided with statistical significance set at *p* < 0.05. Analyses were conducted using StataNow 19.5 (StataCorp LLC, College Station, TX, USA). As the available implementation data were designed primarily to track pathway progression and because several staged outcomes had sparse cells or complete separation, centre-level analyses were treated as exploratory and unadjusted. Multivariable logistic regression was therefore not used for causal inference in this formative report; future analyses with a fuller individual-level implementation dataset should examine predictors of contact success, T0 completion, and attrition.

## 3. Result

**Figure 1:**
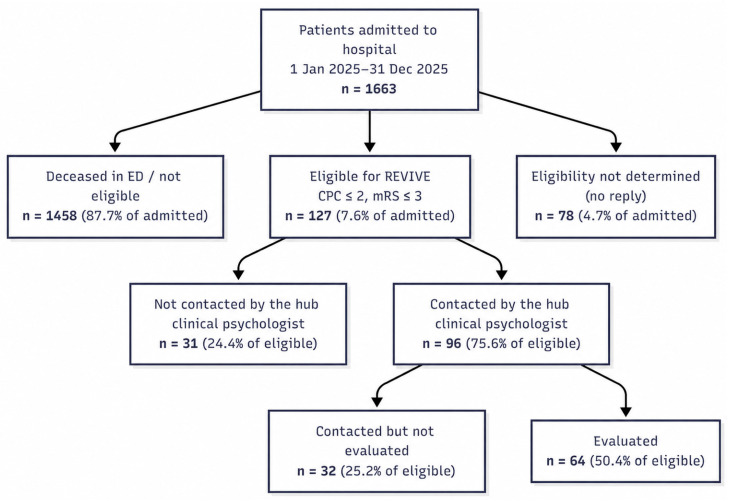
Flow diagram of screening, eligibility, contact, and evaluation within the REVIVE survivorship follow-up pathway during its first 12 months. Percentages refer to admitted patients at the screening stage and to eligible patients thereafter. CPC = Cerebral Performance Category; mRS = Modified Rankin Scale; ED = emergency department.

Figure 1 illustrates the cohort flowchart for REVIVE’s first 12 months (January–December 2025).

Of the 1663 patients who arrived at the hospital, 1458 (87.6%) were recorded as deceased or having an unfavourable neurological outcome (CPC 3 or 4) and were therefore outside the intended REVIVE target population. Among the remaining 205 patients, 78 (4.7% of the total cohort) had insufficient information to determine eligibility, while 127 (7.6% of the total cohort) met protocol eligibility criteria. Of these 127 eligible patients, 31 (24.4%) were not contacted, for the reasons listed in Table 1. In particular, there was no contact information for 21 individuals, and 8 refused enrolment at the local hospital. The hub team contacted 96 patients; of these, 64 (50.4% of eligible patients; 66.7% of contacted patients) completed the T0 assessment, whereas 32 did not proceed with the assessment. The main reasons for refusal among contacted patients are detailed in Table 2, with non-response/unreachability (n = 11, 8.6% of eligible) and personal refusal/lack of interest (n = 8, 6.3%) accounting for the majority.

Of the eligible patients (Table 3), the majority were men (n = 102; 80.3%) with median age 63 years (IQR: 54–73).

Of 1663 screened OHCA patients, observed eligibility rates (eligible/total screened) were higher in the Pavia hub (26/133, 19.5%) than in spoke centres (101/1530, 6.6%; *p* < 0.001; Table 3), Pavia vs. other university centres (26/133, 19.5% vs. 34/344, 9.9%; *p* = 0.004; Table 4), and university vs. non-university centres (60/477, 12.6% vs. 67/1186, 5.6%; *p* < 0.001; Table 5). These comparisons are reported as unadjusted implementation indicators and should not be interpreted as evidence that centre type causes differences in pathway performance.

Among the 127 eligible patients, observed contact rates (contacted/eligible) were higher in Pavia than in spoke centres (24/26, 92.3% vs. 72/101, 71.3%; *p* = 0.026; Table 3), and numerically higher in Pavia than in other university centres (24/26, 92.3% vs. 25/34, 73.5%; *p* = 0.093; Table 4). Contact rates did not differ significantly between university and non-university centres (49/60, 81.7% vs. 47/67, 70.1%; *p* = 0.13; Table 5).

Among the 96 contacted patients, assessment completion (assessed/contacted) was higher in Pavia than in spoke centres (24/24, 100% vs. 40/72, 55.6%; *p* < 0.001; Table 3), and higher in Pavia than in other university centres (24/24, 100% vs. 13/25, 52.0%; *p* < 0.001; Table 4), with a non-significant difference between university and non-university centres (37/49, 75.5% vs. 27/47, 57.4%; *p* = 0.061; Table 5). Given the complete assessment among contacted Pavia patients and the absence of adjustment for staffing, workflow, local infrastructure, and patient mix, these differences should be viewed as exploratory signals of implementation capacity.

## 4. Discussion

The REVIVE project represents the first structured survivorship pathway for out-of-hospital cardiac arrest (OHCA) survivors in the Lombardy region, implementing a hub-and-spoke model that coordinates multidisciplinary follow-up across multiple hospitals [8]. The present findings reflect a cohort comprised mainly of survivors with favourable neurological recovery and sufficient capacity to engage in follow-up, and therefore may not fully capture the needs of OHCA survivors with more complex rehabilitation trajectories. The predominance of men among eligible survivors in REVIVE (80.3%) is consistent with wider OHCA literature describing important sex differences in incidence and outcomes [10]. This pathway delivered structured T0 assessment for 64 of the 127 eligible survivors, corresponding to 50.4% of the intended target group. Although current results indicate that almost half of the eligible survivors did not complete the first assessment, the value of this early evaluation lies in identifying where losses occurred across the pathway and which implementation processes require optimisation before claims about feasibility, sustainability, or survivor outcomes can be made. These findings suggest that the pathway could be initiated and delivered across multiple centres; however, longer-term feasibility, sustainability, and acceptability remain to be established. Acceptability signals were also mixed, with low in-hospital refusal but substantial post-discharge non-response, lack of interest, and perceived well-being. The first 12 months of REVIVE here reported should therefore be interpreted as formative implementation data, not as evidence of a mature or clinically effective survivorship service.

### 4.1. Barriers and Improvement Opportunities

Several implementation barriers limited pathway coverage, particularly at the identification and contact stages. The first barrier was the incomplete capture and transfer of key pathway-entry information. Eligibility status remained unknown for 78 patients (4.7%), likely reflecting a combination of incomplete screening documentation, the missing transfer of key information, and limited local capacity for systematic case identification in some smaller spoke hospitals. Among the 127 eligible patients, 21 (16.5%) could not be contacted because telephone numbers or email addresses were missing. In the context of a prospective formative evaluation, these findings highlight early weaknesses in data collection and information transfer that affected pathway entry [11].

Potential strategies for improvement at this stage centre on strengthening discharge and referral processes. Previous implementation studies suggest that structured discharge checklists can reduce data loss and improve follow-up rates [7]; they could therefore be used to ensure the systematic transfer of survivor contact details from peripheral hospitals to the central hub, and may therefore improve pathway coverage [12]. Use of the Lombardia CARe registry, together with reminders sent by the hub team, could further reduce these early identification and contact gaps.

A second major implementation barrier was attrition after discharge. While in-hospital refusal remained low at 6.3% (n = 8), attrition increased after hub contact: of the 96 patients reached post-discharge, 32 (33.3%) did not complete the T0 assessment. The most common reasons were non-response or unreachability (n = 11, 8.6%), personal refusal or a lack of interest (n = 8, 6.3%), perceived well-being (n = 3), emotional overload (n = 2), and language or logistical barriers (n = 2). This pattern suggests that some survivors may not perceive follow-up as necessary once acute recovery appears satisfactory. This is consistent with previous reports showing that loss to follow-up among cardiac arrest survivors remains a challenge across 3-, 6-, and 12-month follow-up windows [13]. Although multivariable predictors were not examined in this study, the observed patterns suggest that successful contact and assessment completion were likely influenced by the completeness of contact information and by centre-level organisational differences. Attrition after contact was mainly associated with non-response, lack of interest, perceived well-being, emotional overload, and logistical barriers. These factors warrant further study in future analyses using a fuller dataset.

Potential strategies to address this barrier should therefore focus on engagement; psychoeducation during admission may help counter the underestimation of long-term cognitive, psychological, and functional difficulties and reinforce the purpose of follow-up [14]. Flexible contact options and brief educational materials may further reduce disengagement [15,16].

A third implementation barrier concerned structural and organisational differences between centres. Pavia showed higher observed rates of eligibility ascertainment, patient contact, and assessment completion; however, these differences were unadjusted and may reflect staffing, research infrastructure, direct access to the coordinating team, workflow familiarity, patient mix, and local organisational culture. Pavia should therefore be interpreted as a specific case of embedded implementation capacity, not as proof that the hub model is intrinsically superior to spoke delivery. The relevant implementation question is how the functions that appeared to work well in Pavia, including reliable identification, contact transfer, scheduling, and assessment capacity, can be reproduced or supported across spoke centres.

Potential strategies for improvement at the organisational level include greater procedural standardisation, clearer allocation of responsibilities, and targeted operational support for spoke centres with fewer staff or less research infrastructure. These strategies should be evaluated in future implementation cycles rather than assumed to resolve centre-level variation.

A fourth implementation barrier was logistical difficulty within the hub-and-spoke model itself. Patients managed at spoke centres who lived far from the central hub, particularly older patients and those with limited access to or confidence with technology, may have faced additional barriers to attending in-person assessments.

Potential strategies for overcoming these logistical barriers include more flexible hybrid delivery models, such as local assessments supported by video link, simplified telephone-first pathways, and family-supported digital access [13,17]. Such approaches may help preserve hub expertise while reducing barriers related to travel distance and digital exclusion.

### 4.2. Facilitators

Several potential facilitators were identified, but these should be understood as plausible implementation enablers observed through pathway tracking rather than formally tested mechanisms.

A core feature was the centralisation of specialised assessments and coordination at Pavia hub while maintaining local continuity at spoke centres. This configuration provided a mechanism through which survivorship care could be coordinated without requiring each hospital to establish its own independent multidisciplinary team.

The introduction of Lombardy’s first structured post-OHCA survivorship program may also have increased the visibility of survivorship needs among participating clinicians. In a context where post-discharge cardiology contacts had often been fragmented, REVIVE introduced a more formalised and evidence-informed follow-up pathway. This may have encouraged engagement from spoke centres, but it should be regarded as an early implementation observation rather than evidence that the model is established as a regional benchmark.

The hub-spoke operational model also appeared to support pathway delivery. Dedicated local referents at each spoke hospital, reinforced by weekly hub reminders, helped distribute responsibility across sites. Spoke coordinators integrated patient screening into routine workflows, such as ward rounds and discharge planning. Hub oversight may have reduced the risk of eligible survivors being missed between discharge from the spoke site and first contact, although this requires further evaluation.

Flexible assessment modalities, including in-person at the Pavia hub, telephone, or videoconference, also appeared to support uptake. This approach may also have facilitated participation by accommodating mobility limitations, fatigue, travel burden, and variation in digital literacy, particularly among older patients at spoke sites.

A multidisciplinary team comprising cardiologists, psychologists, and coordinators enabled follow-up across cardiac, cognitive, and psychological domains, extending beyond a cardiology-only model. Regular team case reviews also facilitated real-time adaptation, including the management of individual barriers such as rescheduling in the context of emotional overload, and iterative refinement of the protocol during implementation.

Prospective database tracking provided detailed visibility of pathway progression. Each stage, including identification, consent, assessment, and reasons for attrition, was recorded systematically. This enabled the closer monitoring of implementation and informed practical adjustments, such as intensified reminders for slower-responding spoke sites or changes in modality based on uptake patterns. Within Proctor et al.’s framework, these data primarily inform reach/penetration, feasibility, fidelity, and acceptability; they do not yet establish appropriateness, cost, sustainability, or clinical effectiveness.

## 5. Conclusions

REVIVE should be interpreted as an early formative implementation of a structured hub-and-spoke survivorship pathway, not as evidence of established effectiveness or mature feasibility during the first year, the pathway enabled structured T0 assessment for 64 of 127 eligible survivors; it, but also revealed substantial attrition between eligibility, contact, and assessment. The 50.4% eligible-to-assessed proportion is therefore best understood as an initial internal implementation indicator against which future optimisation cycles can be assessed.

Potential priorities for future optimisation include standardised discharge and contact-information checklists, earlier explanation of the purpose of follow-up, clearer allocation of spoke-site responsibilities, and flexible assessment models that preserve hub expertise while reducing travel and digital-access barriers. Future evaluations should examine patient and clinician acceptability, sustainability, costs, and clinical outcomes once the implementation pathway is more stable.

## Figures and Tables

**Table 1:** Reasons why patients were not contacted by the hub centre.

Reasons for Non-Contact by Hub Centre	Patients, n (% Eligible)
No contact information	21 (16.5%)
In hospital refusal	8 (6.3%)
No callback	1 (0.8%)
Transfer to another hospital outside network	1 (0.8%)

**Table 2:** Reasons why patients refused to participate.

Reason Provided for Refusal	Patients, n (% Eligible)
No response/Non contactable	11 (8.6%)
Not interested	8 (6.3%)
Refusal by proxy (family member)	3 (2.4%)
Already under care elsewhere	3 (2.4%)
Perception of well-being	3 (2.4%)
Overload	2 (1.6%)
Logistical barriers	2 (1.6%)

**Table 3:** **Comparison between Pavia and non-Pavia centres.** Data are presented as n (%). Percentages for contact status were calculated among eligible patients; percentages for assessment status were calculated among contacted patients.

Outcome	Response	Non-Pavia (n = 1530)	Pavia (n = 133)	Total (n = 1663)	Test	*p*-Value
**Eligibility status**	No	1351 (88.3%)	107 (80.5%)	1458 (87.7%)	χ^2^(2) = 34.50	<0.001
	Yes	101 (6.6%)	26 (19.5%)	127 (7.6%)		
	Not determined	78 (5.1%)	0 (0.0%)	78 (4.7%)		
**Contact status**	No	29 (28.7%)	2 (7.7%)	31 (24.4%)	χ^2^(1) = 4.95	0.026
	Yes	72 (71.3%)	24 (92.3%)	96 (75.6%)		
**Assessment status**	No	32 (44.4%)	0 (0.0%)	32 (33.3%)	χ^2^(1) = 16.00	<0.001
	Yes	40 (55.6%)	24 (100.0%)	64 (66.7%)		

**Table 4:** **Comparison between other university centres (Other Uni) and Pavia (PV).** Data are presented as n (%). Percentages for contact status were calculated among eligible patients; percentages for assessment status were calculated among contacted patients.

Outcome	Response	Other Uni (n = 344)	PV (n = 133)	Total (n = 477)	Statistical Test	*p*-Value
**Eligibility status**	No	302 (87.8%)	107 (80.5%)	409 (85.7%)	Fisher’s exact	0.004
	Yes	34 (9.9%)	26 (19.5%)	60 (12.6%)		
	Not determined	8 (2.3%)	0 (0.0%)	8 (1.7%)		
**Contact status**	No	9 (26.5%)	2 (7.7%)	11 (18.3%)	Fisher’s exact	0.093
	Yes	25 (73.5%)	24 (92.3%)	49 (81.7%)		
**Assessment status**	No	12 (48.0%)	0 (0.0%)	12 (24.5%)	Fisher’s exact	<0.001
	Yes	13 (52.0%)	24 (100.0%)	37 (75.5%)		

**Table 5:** **Comparison between non-university centres (Not Uni) and University centres (Uni).** Data are presented as n (%). Percentages for contact status were calculated among eligible patients; percentages for assessment status were calculated among contacted patients.

Outcome	Response	Not Uni (n = 1186)	Uni (n = 477)	Total (n = 1663)	Statistical Test	*p*-Value
**Eligibility status**	No	1049 (88.4%)	409 (85.7%)	1458 (87.7%)	Fisher’s exact	<0.001
	Yes	67 (5.6%)	60 (12.6%)	127 (7.6%)		
	Not determined	70 (5.9%)	8 (1.7%)	78 (4.7%)		
**Contact status**	No	20 (29.9%)	11 (18.3%)	31 (24.4%)	Fisher’s exact	0.13
	Yes	47 (70.1%)	49 (81.7%)	96 (75.6%)		
**Assessment status**	No	20 (42.6%)	12 (24.5%)	32 (33.3%)	Fisher’s exact	0.061
	Yes	27 (57.4%)	37 (75.5%)	64 (66.7%)		

## Data Availability

Data are available upon reasonable request to the authors.
